# Linking factor XIII activity to all-cause mortality after myocardial infarction: the overlooked role of serum albumin

**DOI:** 10.1016/j.ijcha.2025.101796

**Published:** 2025-09-11

**Authors:** Jan Traub, Makram Abu Hussein, Dominik Schmitt, Anna Frey

**Affiliations:** a Department of Internal Medicine I, University Hospital Würzburg, Germany; b Comprehensive Heart Failure Center, University and University Hospital Würzburg, Germany

**Keywords:** Myocardial infarction, Factor XIII, Albumin, Prognosis, Mortality, Biomarkers

## Abstract

**Background:**

Acute myocardial infarction (MI) remains a major cause of morbidity and mortality despite therapeutic advances. Factor XIII (FXIII), a fibrin-stabilizing enzyme with roles in coagulation, inflammation, and tissue repair, has emerged as a potential biomarker in MI. While low FXIII activity has been linked to adverse outcomes, the underlying determinants and its independent prognostic value remain unclear.

**Methods:**

In this retrospective study, FXIII activity was measured in 926 MI patients treated at University Hospital Würzburg between 2018 and 2023. Blood samples were collected within 24 h of cardiac catheterization. FXIII activity was assessed photometrically, and patients were followed for all-cause mortality. Multivariable regression and Cox models were used to identify predictors of FXIII activity and mortality.

**Results:**

Median FXIII activity was 110 %. Lower FXIII activity was associated with older age, female sex, lower albumin, higher CRP, and reduced kidney function. While crude mortality at 30 days and 1 year was significantly higher in patients with FXIII ≤ 110 %, FXIII activity was not an independent predictor of mortality after adjustment. Key predictors included albumin (HR = 0.221, p < 0.001), age (HR = 1.048, p < 0.001), eGFR (HR = 0.988, p = 0.001), and ASAT (HR = 1.001, p = 0.002).

**Conclusions:**

Although lower FXIII activity is associated with higher mortality post-MI, this effect is largely mediated by albumin levels. Albumin appears to be a central determinant of both FXIII activity and prognosis, highlighting its potential role as a key marker in risk stratification. Further studies are warranted to explore therapeutic implications of hypoalbuminemia in MI.

## 1 Introduction

Acute myocardial infarction (MI) is a leading cause of morbidity and mortality globally, posing a significant public health challenge. Despite interventional and medicamentous advancements, the long-term prognosis remains poor due to manifold short- and long-term complications, underscoring the need for improved treatment strategies beyond immediate intervention.

Within the recent years, factor XIII (FXIII), a transglutaminase enzyme, has gained attention in the MI. In the coagulation cascade, FXIII acts downstream of thrombin: once fibrinogen is converted to fibrin by thrombin, FXIII is cleaved and activated to FXIIIa. Activated FXIII then crosslinks fibrin strands, stabilizing the clot and protecting it from fibrinolysis. This places FXIII at the final step of the coagulation process, linking thrombin generation to clot stabilization. Beyond its role in coagulation, FXIII is involved in inflammation regulation and tissue repair [1]. First emerging evidence suggests that lower FXIII activity after MI indeed associates higher overall mortality [2] and cardiac remodeling [3]. Murine studies showed beneficial effects of XIII supplementation in knock-out animals [4,5], indicating a potential therapeutic option in humans also. However, determinants of FXIII activity and cofounders of its effect on mortality after MI remain uncertain.

## 2 Methods

Against this backdrop, FXIII activity was quantified in most MI patients at the University Hospital of Würzburg between 2018 and 2023. FXIII activity was measured photometrically on a BCS-XP analyzer (Siemens Healthineers, Germany; catalog no. OTXA15), where plasma FXIII is activated by thrombin to FXIIIa and quantified via ammonia release and coupled enzymatic reaction measured at 340 nm, thereby reflecting the activation capacity of the circulating zymogen. Blood samples (citrate plasma) were taken within 24 h before or after the cardiac catheterization, along with routine laboratory parameters. Albumin concentrations were measured using the bromcresol green colorimetric endpoint assay (Cobas analyzer, Roche), which quantifies total serum albumin without distinction between oxidized and non-oxidized fractions. Resident registration offices were contacted in summer 2024 to obtain overall mortality data, without specification of cause of death. In this large retrospective analysis, which was approved by the local ethics board (#20230618–01), we aimed to identify independent predictors of both FXIII activity and all-cause mortality after MI.

## 3 Results

Median age of 926 included MI patients was 64 years (interquartile range 55–74 years) and 216 patients (23 %) were female (Table 1). The majority of 525 patients (57 %) suffered an ST elevation MI, while 401 patients (43 %) had a non-ST elevation MI. The culprit vessel varied among patients, with the left anterior descending artery being the most common at 404 patients (44 %), followed by the right coronary artery in 322 patients (35 %), and the circumflex artery in 163 patients (18 %). Coronary intervention was performed in 757 patients (82 %), with drug-eluting stents used in 635 patients (84 % of those undergoing intervention), bare metal stents in 114 patients (15 %), and drug-eluting balloons in 8 patients (1 %).

**Table 1:** Baseline characteristics of the study sample.

	All patients	FXIII activity ≤110 %	FXIII activity >110 %	P-value
Number of patients	926 (100)	467 (50)	459 (50)	
Age (years)	64 (55–74)	67 (56–76)	61 (54–69)	**<0.001**
Female sex	216 (23)	96 (21)	120 (26)	**0.044**
ST elevation myocardial infarction	525 (57)	244 (52)	281 (61)	**0.006**
Non-ST elevation myocardial infarction	401 (43)	223 (48)	178 (39)	**0.006**
Culprit vessel				
- Main stem	36 (4)	25 (5)	11 (2)	**0.020**
- Left anterior descending artery	404 (44)	211 (45)	193 (42)	0.336
- Circumflex artery	163 (18)	85 (18)	78 (17)	0.629
- Right coronary artery	322 (35)	151 (32)	171 (37)	0.116
- Bypass vessel	15 (2)	10 (2)	5 (1)	0.205
- None	63 (7)	39 (8)	24 (5)	0.059
In-stent stenosis	15 (2)	8 (2)	7 (1)	0.821
Coronary intervention	757 (82)	367 (79)	390 (85)	**0.012**
- Drug-eluting stenting	635 (84*)	300 (82*)	335 (86*)	0.120
- Bare metal stenting	114 (15*)	63 (17*)	51 (13*)	0.116
- Drug eluting balloon	8 (1*)	4 (1*)	4 (1*)	0.931
- Successful intervention	753 (100*)	364 (99*)	389 (100*)	0.287
Number of stents	1 (1–2)	1 (1–2)	1 (1–2)	0.652
Coronary artery bypass graft indication	70 (8)	38 (8)	32 (7)	0.502
Highest creatine kinase (U/l)	771 (285–1660)	755 (251–1920)	797 (306–1764)	0.051
Albumin (g/dl)	4.0 (3.7–4.2)	3.8 (3.4–4.1)	4.1 (3.9–4.3)	**<0.001**
C-reactive protein (mg/dl)	0.6 (0.2–2.1)	0.8 (0.2–3.6)	0.5 (0.2–1.3)	**<0.001**
Alanine transferase (U/l)	37 (23–65)	41 (24–75)	34 (23–55)	**0.040**
Asparate transferase (U/l)	93 (40–218)	105 (41–256)	82 (27–182)	**0.007**
Estimated glomerular filtration rate (ml/min/1.73 m^2^)	81 (64–95)	79 (59–94)	82 (67–96)	0.078
International normalized ratio	1.06 (1.00–1.15)	1.07 (1.01–1.18)	1.05 (0.99–1.13)	**<0.001**
Previous myocardial infarction	102 (11)	65 (14)	37 (8)	**0.006**
Previous stroke	19 (2)	11 (2)	8 (2)	0.670
Previous transient ischemic attack	8 (1)	5 (1)	3 (1)	0.725
Arterial hypertension	688 (74)	338 (72)	350 (76)	0.203
Atrial fibrillation	153 (17)	94 (20)	59 (13)	**0.004**
Heart failure	427 (46)	223 (48)	204 (44)	0.345
Peripheral artery disease	36 (4)	23 (5)	13 (3)	0.140
Diabetes mellitus	236 (25)	142 (30)	94 (20)	**<0.001**
Chronic kidney disease	517 (56)	257 (55)	260 (57)	0.669
Liver disease	34 (4)	22 (5)	12 (3)	0.128
30-days mortality	86 (9)	61 (13)	25 (5)	**<0.001**
1-year mortality	115 (12)	81 (17)	34 (7)	**<0.001**

Data are median (quartiles) or count (percentage), as appropriate. Groups were compared by chi-square test or T test. Bold P-values indicate significance (P < 0.005). * Percent of patients with intervention.

FXIII activity ranged from 35 % to 156 %, with a median of 110 % (interquartile range 92–131 %). Patients were divided into two groups based on the median FXIII activity (Table 1). Patients with FXIII activity ≤ 110 % were older, more likely to be female and had higher rates of main stem involvement and bare metal stenting. They also had higher alanine transferase (ALAT), higher aspartate transferase (ASAT), lower albumin, and higher C-reactive protein (CRP) levels, as well as slightly higher international normalized ratio (INR). Maximal creatine kinase (CK) did not differ between groups. Baseline comorbidities derived from ICD to 10 codes showed distinct differences between patients with FXIII activity ≤110 % and >110 %, with higher rates of previous myocardial infarction, atrial fibrillation and diabetes mellitus in the lower FXIII group (Table 1). FXIII activity was not significantly correlated with the timing of blood sampling relative to catheterization (Spearman correlation, p = 0.216), indicating that measurements were independent of whether samples were drawn before or after reperfusion.”

In a linear multivariable regression model, significant independent associations with FXIII activity were found for albumin (t = 13.2, p < 0.001), gender (female, t = 4.8, p < 0.001), age (t = −3.7, p < 0.001), ALAT (t = 2.8, p = 0.005), eGFR (t = −2.7, p = 0.008), CRP (t = −2.3, p = 0.020), and ASAT (t = −2.4, p = 0.016). Thus, lower FXIII activity mainly associated with lower albumin levels, male gender, and older age. Variables such as maximal CK, type of MI, and INR were not significantly associated with FXIII activity. The adjusted R^2^ for the overall model was 0.27, indicative of a high goodness-of-fit according to Cohen.

**Fig. 1:**
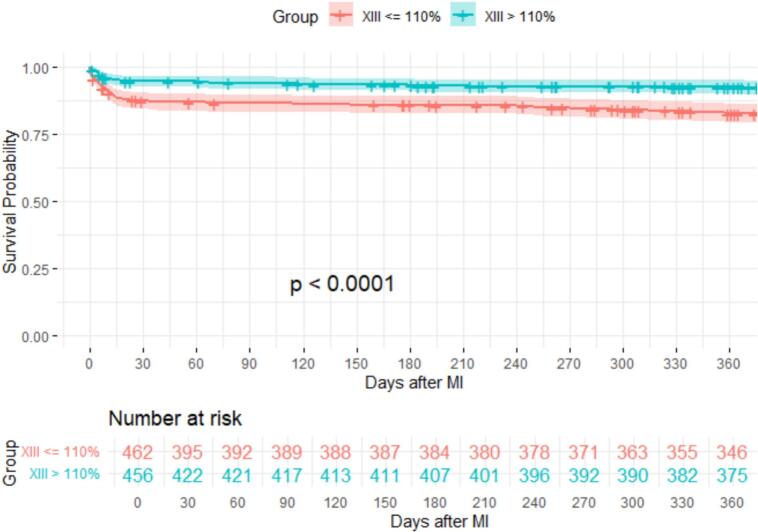
Kaplan Meier Plot of Overall Survival after Myocardial Infarction. Groups according to factor XIII activity (%). MI = myocardial infarction. P value from Log-Rank Test.

Median follow-up time of this cohort was 2.2 (1.1–6.5) years. Significantly higher 30-day (13 % vs. 5 %, p < 0.001) and 1-year mortality (17 % vs. 7 %, p < 0.001) were observed in the FXIII ≤110 % group compared to those with FXIII activity >110 % (Table 1; Fig. 1). However, in a multivariable Cox regression model adjusting for age, gender, albumin, maximal CK, CRP, eGFR, GOT, ALAT, and type of MI, FXIII activity was not a significant predictor of mortality (HR = 1.001 [95 % CI: 0.994–1.008], p = 0.714). Significant predictors of mortality included:

• Albumin: HR = 0.221 [95 % CI: 0.156–0.314], z = −8.490, p < 0.001
• Age: HR = 1.048 [95 % CI: 1.034–1.063], z = 6.628, p < 0.001
• eGFR: HR = 0.988 [95 % CI: 0.982–0.994], z = −4.023, p = 0.001
• ASAT: HR = 1.001 [95 % CI: 1.000–1.001], z = 3.081, p = 0.002

These findings suggest that while FXIII activity alone does not independently predict mortality, other clinical factors, particularly albumin, age, and eGFR, play a critical role in determining patient outcomes. The adjusted model had a high goodness-of-fit, with an adjusted R^2^ of 0.27, and a concordance index of 0.846, indicating strong predictive accuracy.

## 4 Discussion

Our findings align with earlier studies reporting that lower FXIII activity is associated with higher mortality post-MI [2,4]. However, representing the main finding of this study, that when adjusting for other clinical factors (especially albumin levels), FXIII activity alone does not significantly predict mortality. As we could show that FXIII activity is particularly strongly affected by albumin levels, we claim that the association of FXIII activity with mortality is an epiphenomenon, with albumin levels as main mediator.

Given that FXIII is activated by thrombin, the marked thrombin burst occurring during coronary thrombosis and again at reperfusion could substantially convert circulating FXIII to FXIIIa before sampling. This might reduce the measurable zymogen pool and thereby contribute to lower FXIII activity values. However, in our study the strong association with albumin suggests that this potential acute consumption does not fully account for the prognostic link, highlighting albumin as the main mediator. Reduced FXIII activity may also impair clot cross-linking and stability, potentially favoring microthrombus formation and distal embolization, which could contribute to adverse outcomes in addition to the albumin-mediated effects observed. Although FXIII activity was not an independent predictor of outcome in our analysis, its close relationship with albumin suggests that it may not serve as a stand-alone biomarker. However, FXIII could still add value as part of a broader biomarker panel, where its interplay with albumin, renal function, and inflammatory markers might refine prognostic assessment and highlight relevant biological pathways. Differences in comorbidities, particularly the higher prevalence of previous myocardial infarction, atrial fibrillation, and diabetes mellitus in patients with lower FXIII activity, may contribute to their adverse prognosis but do not fully account for the independent association observed with albumin.

While the close correlation between FXIII activity and albumin levels could suggest a direct binding interaction, existing evidence does not support physiologically relevant binding of FXIII to albumin. Instead, both proteins share hepatic synthesis, which may explain their correlated plasma concentrations. Conditions affecting liver function, such as cirrhosis or malnutrition, may therefore reduce their levels. Hypoalbuminemia is a well-known marker of poor prognosis in various diseases, probably due to its association with malnutrition, inflammation, and advanced disease states [6]. However, albumin might also play a role in the natural history of such diseases due to its antioxidant, anti-inflammatory, and antithrombotic properties. In patients with MI, a Japanese study among 1424 subjects showed that low serum albumin (<3.8 g/dl) one year after discharge associated with long-term adverse outcomes [7]. Our findings expand these findings to albumin levels at the time of MI. Regarding other cardiological conditions, among 1,365,529 adult hospitalizations due to acute heart failure in the United States, patients admitted with concomitant hypoalbuminemia had nearly twice the odds of in-patient mortality than those with normal albumin levels [8]. Further, current evidence points to hypoalbuminemia as a marker of poor outcomes in chronic heart failure [9]. Whether albumin supplementation or nutritional support in general would be beneficial in improving clinical outcomes is not completely clear and should be evaluated in adequately designed studies.

A major strength of our study is the large cohort size and the comprehensive data collection, which included a broad range of clinical and laboratory parameters. This allowed for a robust multivariable analysis and the identification of independent predictors of FXIII activity and mortality.

However, several limitations must be acknowledged. The applied photometric assay quantifies the activation capacity of plasma FXIII zymogen rather than FXIII already activated or consumed in vivo. Therefore, reduced activity values could theoretically also reflect prior enzymatic activation and consumption during MI/reperfusion. However, our multivariable analyses indicate that the observed prognostic association is predominantly mediated by serum albumin rather than by acute FXIII consumption. It should be noted that the applied assay detects total albumin but does not distinguish oxidized from non-oxidized forms. Given that albumin acts as a major antioxidant and is readily modified during ischemia–reperfusion, functional impairment due to oxidation may not be reflected by measured concentrations and could represent an additional mechanism underlying the prognostic role of albumin. The retrospective design may introduce selection bias, and the observational nature of the study precludes the establishment of causality. Additionally, the single-center setting may limit the generalizability of our findings. Another limitation of our study is that mortality type (e.g., thromboembolic events or reinfarction) could not be distinguished, as only all-cause mortality was available from the registration offices. Further, there is a lack of individual-level data on antiplatelet and anticoagulant therapy; however, as all patients were treated for acute myocardial infarction according to contemporary guidelines, it can be assumed that virtually all received aspirin, most received dual antiplatelet therapy, and oral anticoagulation was prescribed as indicated. Future studies should consider multi-center designs and prospective data collection to validate and extend our results, including the assessment of potential therapeutic implications.

## CRediT authorship contribution statement

**Jan Traub:** Writing – original draft, Visualization, Software, Formal analysis, Data curation, Conceptualization. **Makram Abu Hussein:** Writing – review & editing, Resources, Methodology, Investigation. **Dominik Schmitt:** Writing – review & editing, Software, Resources, Formal analysis, Conceptualization. **Anna Frey:** Writing – review & editing, Supervision, Project administration, Conceptualization.

## Funding

This work received public funding from the German Research Foundation (453989101). JT was funded by the Interdisziplinäres Zentrum für Klinische Forschung (IZKF) Wuerzburg (bridging program; Z-3BC/10). AF was also funded by the IZKF Wuerzburg (habilitation grant, E-298, S-517).

## Declaration of competing interest

The authors declare that they have no known competing financial interests or personal relationships that could have appeared to influence the work reported in this paper.
